# Correlative Structural Biology: How to Investigate the Fine Details of Viral Structure

**DOI:** 10.3390/v2010107

**Published:** 2010-01-11

**Authors:** Elizabeth R. Wright

**Affiliations:** Emory University School of Medicine, Department of Pediatrics, Division of Pediatric Infectious Diseases, 2015 Uppergate Drive, NE, Atlanta, GA 30322, USA; E-Mail: erwrigh@emory.edu; Tel.: +1 404-727-4665

## Abstract

Commentary on Byeon, I.J.; Meng, X.; Jung, J.; Zhao, G.; Yang, R.; Ahn, J.; Shi, J.; Concel, J.; Aiken, C.; Zhang, P.; Gronenborn, A.M. Structural convergence between Cryo-EM and NMR reveals intersubunit interactions critical for HIV-1 capsid function. *Cell* **2009**, *139*, 780–790.

In the 13 November 2009 issue of *Cell*, In-Ja Byeon and colleagues from the University of Pittsburgh and Vanderbilt University used a combination of molecular virology, advanced cryo-EM imaging, and high-resolution solution-state NMR techniques to determine the fine structure relationships essential for HIV-1 capsid formation and function [1]. This study is of high interest to virologists and structural biologists alike, as it demonstrates the clear advantage of coordinating research efforts to answer fundamental questions regarding the nature of sub-viral ultrastructure.

Retroviruses, such as the human immunodeficiency virus type 1 (HIV-1), undergo a viral maturation process, which is driven by the proteolytic cleavage of the Gag polyprotein. During maturation, the capsid protein (CA) rearranges to form the ‘capsid,’ an enclosure that protects the viral genome. For retroviruses, the capsid is variable in structure, and HIV-1 generally has a conical capsid. What fascinates many structural biologists and retrovirologists is the ability of the CA protein to rearrange from one hexameric lattice to another and how multiple intermolecular interfaces stabilize the two structures. Three complementary structural methods are generally employed to examine viral ultrastructure to either macromolecular or atomic resolution: cryo-electron microscopy (cryo-EM), NMR, and x-ray crystallography. Each technique enables investigators to gain specific insights into the structure of complex macromolecular assemblies. However, all three methods have inherent limitations when examining viral structure.

One of the major developments to electron microscopy (EM), which propelled forward studies of viral ultrastructure, was cryo-EM. Cryo-EM samples are imaged in the frozen-hydrated state without the introduction of structure altering artifacts associated with sample fixation, dehydration, plastic embedding, and staining. By using this method to image HIV-1 and assemblies of viral proteins (e.g. CA), research groups are able to observe the virus or viral components in their native state at resolutions greater than previously possible. However, one major limitation for the cryo-EM analysis of intact, pleiomorphic viruses such as HIV-1 is the inability to achieve atomic- or near-atomic level resolution of the virus. In order to overcome this constraint, investigators examine well-ordered assemblies of proteins, including CA, and subject them to rigorous computational analysis to generate a composite average 3D reconstruction of the well-ordered assembly. Once density maps are generated, protein domain models from x-ray crystallography or NMR studies may be docked into the map in order to generate a representation of how the individual protein domains interact to form a stable structure.

X-ray crystallography and NMR are used to examine the structure of proteins, nucleic acids, and other biological macromolecules. These methods provide us with the highest resolution (atomic) structures possible; however, each method has associated limitations. With crystallography, the most significant challenge lies in crystallizing the target macromolecule; not every material is able to crystallize. The sample must also be extremely pure and homogeneous so that, under the appropriate conditions, it may crystallize into a uniformly ordered static-array. Solution-state biological NMR also requires the sample to be highly purified and relatively concentrated. An additional constraint for NMR is that it is useful for solving the structures of relatively small macromolecules (e.g., a protein with a molecular weight of less than 100 kDa). However, NMR has a major advantage over x-ray methods in that the sample remains in the aqueous (solution) state, which can be more relevant for certain structural analyses and comparisons.

In an effort to overcome the restrictions of individual technologies and shed light on some of the complexities of retrovirus dis/assembly, research teams at the University of Pittsburgh and Vanderbilt University examined the structure of HIV-1 CA protein assemblies using both cryo-EM and high-resolution NMR. The study reported by Byeon *et al*. describes the detailed analysis of the 16-Å resolution cryo-EM structure of HIV-1 CA tubular assemblies and the correlation with the high-resolution NMR solution-structure of the CA CTD dimer. This study is positioned to become a benchmark for the successful merger of cryo-EM and solution-state NMR examinations of viral proteins. Two major advances were that the primary cryo-EM data of CA protein tubular assemblies was obtained and analyzed at a higher resolution than previously attained, and the solution-state NMR structure of the CA CTD dimer was the first to have been reported.

First, the authors examined the arrangement of the whole CA lattice structure through cryo-EM studies of full-length HIV-1 CA tubular assemblies. They noted that a number of variable morphologies were generated with the in vitro assembly reactions. The wild-type (WT) HIV-1 CA produced heterogeneously structured tubes (Figure 1a); therefore the authors used a CA mutant (CA A92E), first reported by Li *et al.* [2], that produced individual, long, well-ordered tubes (Figure 1b). By using this construct for cryo-EM data collection and subsequent computational analysis, the authors increased their likelihood of achieving a high-resolution structure. Analysis of the tubes revealed that the tube’s dimensions were consistent with previous reports in the literature [2–3].

As a next step, to evaluate the CTD interactions at higher resolution, the authors used solution-state NMR techniques. The group used multiple complex 2D and 3D NMR experiments to assign the backbone and side chain resonances, and to determine the distance constraints within the CTD dimer. The NMR findings expanded upon a number of studies that have been aimed at illuminating the structure of the intermolecular interfaces that regulate the stability of the CA protein hexameric lattice [4–5]. Their first key finding was that, in solution, only the CTD was responsible for dimerization and the interface is distinguished by hydrophobic interactions formed between residues of the N terminus qof the CTD and of helix 9. They specifically identified Tyr145 as being critical to the interface and successful capsid and particle assembly. Additional EM analysis of Tyr145 mutants supports their NMR findings (supplementary data). The authors surmise that it is perhaps the maintenance of the CTD dimer interface and the plasticity of the others that allows the capsid of HIV-1 and other retroviruses to maintain structural variability. Their second major finding was that the solution CTD dimer fit into the full-length CA structure formed from 2D sheets [4], which was not the case for the crystal structure. This was proposed to be due to preservation of the solution interface.

A final pseudoatomic model generated from the CA tubular assemblies and additional mutational analyses, brought greater clarity to residues involved in the new CTD-CTD interface located at the three-fold axis (Figure 2a and b). From the model, several functionally important residues contained within two helices (H10 and H11) of the CTD dimer interface were re-examined for their impact on infectivity (e.g. capsid stability). Double-cysteine mutations to residues K203/Q219 and P207/T216 resulted in the production of noninfectious particles or reduced infectivity, respectively. This evidence points to the requirement of this newly identified three-fold axis of the CTD-CTD dimer for assembly of the functional capsid of HIV-1. In closing, this study further represents the growing need for the formation of collaborative, interdisciplinary research teams to successfully examine complex questions associated with how structure ultimately governs viral function.

## Figures and Tables

**Figure 1.:**
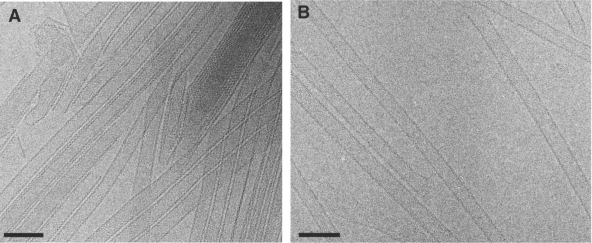
Cryo-EM images of tubular assemblies of recombinant wild-type (WT) HIV-1 CA **(A)**, and HIV-1 CA A92E **(B)**. Scale bars, 100 nm. Reprinted from [1], with permission from Elsevier.

**Figure 2.:**
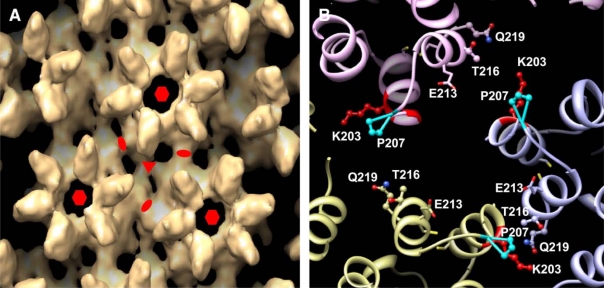
Highlights of the three-fold axis interhexamer interface of the CA CTD dimer. **(A)** Surface rendering of the EM density map obtained from the CA tubular assemblies. The symmetry axes are indicated as follows: two-fold (ellipse), three-fold (triangle), and six-fold (hexagon). **(B)** Detail view of the three-fold axis. Residues known to be associated with the interface are labeled. Reprinted from [1], with permission from Elsevier.
